# Antidiabetic Drug Associations With Heart Failure Outcomes: Real-World Evidence Study Using Electronic Health Records

**DOI:** 10.2196/85083

**Published:** 2026-04-15

**Authors:** Elzbieta Jodlowska-Siewert, Yunhui Chen, Sinian Zhang, Jia Li, Robert Dellavalle, Rui Zhang, Jue Hou

**Affiliations:** 1 Division of Biostatistics and Health Data Science, School of Public Health, University of Minnesota, 2221 University Ave SE, Suite 200, Minneapolis, MN, 55414, United States, 1 612-624-4655; 2 School of Statistics, College of Liberal Arts, University of Minnesota, Minneapolis, MN, United States; 3 Department of Surgery, Division of Computational Health Sciences, Medical School, University of Minnesota, Minneapolis, MN, United States; 4 Department of Dermatology, Medical School, University of Minnesota, Minneapolis, MN, United States

**Keywords:** comparative effectiveness, natural language processing, glucagon-like peptide-1 receptor agonists, dipeptidyl peptidase-4 inhibitors, sulfonylureas, insulin

## Abstract

**Background:**

Patients with type 2 diabetes mellitus (T2D) have a higher risk of cardiovascular disease, including heart failure (HF), leading to health care burden including hospitalization and mortality. Among multiple T2D therapies, there are inadequate head-to-head comparisons of their effects on HF in the real-world patient population.

**Objective:**

This study aims to compare the time-to-HF among patients treated with different T2D drugs following metformin.

**Methods:**

We conducted a retrospective data analysis on electronic health records of 5000 patients with T2D. The inclusion criteria were previous treatment with metformin and initiation of glucagon-like peptide-1 receptor agonists (GLP1 RAs), dipeptidyl peptidase-4 inhibitors (DPP4i), sulfonylureas, or insulin. We grouped patients by the mechanism of their subsequent therapies and focused on 2 pairs of comparisons classified by insulin resistance: sulfonylureas versus insulin (increased resistance) and GLP1 RA versus DPP4i (decreased resistance). The outcomes were 5-year HF status and the HF-free survival time, which was verified manually by examining clinical notes. We applied doubly robust causal estimation and accounted for confounding by adjusting for coded and natural language processing electronic health record features identified through medical knowledge networks.

**Results:**

The study included 939 patients, of whom 204 (21.7%) received insulin, 482 (51.3%) received sulfonylureas, 90 (9.6%) received GLP1 RA, and 163 (17.4%) received DPP4i. Patients in the sulfonylureas group had a significantly higher 5-year HF-free survival compared to the insulin group (survival ratio of insulin/sulfonylureas 0.902, 95% CI 0.840‐0.976; *P=*.01). There was no significant difference between the DPP4i versus GLP1 RA group in 5-year HF-free survival (survival ratio of GLP1 RA/DPP4i was 0.953, 95% CI 0.849‐1.067; *P=*.40). For the occurrence of a HF-related hospitalization within 5 years, there were no significant differences between the sulfonylureas and insulin groups (risk difference 0.057, 95% CI –0.011 to 0.132; *P=*.11), and between the GLP1 RA and DPP4i groups (risk difference 0.010, 95% CI –0.096 to 0.129).

**Conclusions:**

We evaluated real-world evidence on 2 head-to-head comparisons of second-line T2D therapies on 5-year HF outcomes. Patients on sulfonylureas were associated with lower 5-year HF risks than those treated with insulin when measured by risk ratio, but no significant difference was detected when measured by the risk difference. Limitations of this study included potentially inadequate adjustment of confounding in the observational study and a limited sample size with validated HF outcomes.

## Introduction

Diabetes mellitus is a chronic disease caused by impaired glucose metabolism [1]. In 2021, more than 38 million US adults (14.7%) had diabetes, with an additional 8.7 million (3.4%) people who remained undiagnosed. It was estimated that 97.6 million people lived with prediabetes [2], with type 2 diabetes (T2D) accounting for 90% of all cases [3]. Patients with T2D have a 2 to 4 times higher risk of cardiovascular diseases (CVDs), and on average develop CVDs almost 15 years sooner than nondiabetic individuals [4,5], leading to morbidity and mortality [6]. Heart failure (HF) is one of the most expensive CVDs in patients with T2D [7], because HF exacerbations are a common cause of hospitalization [8-10]. Five-year HF-related hospitalization rates in individuals with T2D vary between 3% and 44% [11-13]. Furthermore, HF has the highest 30-day rate of rehospitalizations, with up to 25% of readmissions caused by HF exacerbation [14]. Therefore, control of HF-related hospitalizations is an important goal for T2D management [9,14].

Several medications have been developed and approved for treating T2D, which are often grouped by their mechanisms of action, including glucagon-like peptide-1 receptor agonists (GLP1 RA), dipeptidyl peptidase-4 inhibitors (DPP4i), and sodium-glucose cotransporter 2 inhibitors (SGLT2i). Due to its availability, affordability, effectiveness, and side effect profile, metformin has long been a typical first-line treatment up until recently and still remains highly recommended [15]. Some of these medications do not solely target short-term glycemic control but also improve long-term outcomes such as obesity and lower risks of CVDs [16,17]. As traditional treatment options, insulin and sulfonylureas are still widely used for their availability, affordability, and effectiveness in glycemic control when other treatments fall short [18]. Among the treatments, SGLT2i has demonstrated cardiovascular benefits on HF outcomes in clinical trials and is now used to treat HF [19-21]. There is evidence that some GLP1 RA drugs improve symptoms of HF in patients with obesity with preserved ejection fraction [22], while some DPP4i drugs may increase HF-related hospitalization rates [23], but there is no proven class effect in all patients with HF for any other T2D medication [24]. Electronic health records (EHRs) provide a data source for real-world evidence [25-28] when direct evidence from clinical trials is absent [29]. As part of routine care, EHRs document medical notes and codes for administrative and billing purposes, which also contain necessary information for clinical variables, such as diagnoses, prescriptions, procedures, laboratory tests, and vital signs. While filtering for study-related information from large databases can be challenging [30], recent development of knowledge networks extracted from longitudinal EHRs now provides a means for effective and efficient confounding variable selection [31,32]. For a key clinical variable, such as the HF outcomes, studies have reported discrepancies between disease onset status and apparent EHR codes matched by description [33-36]. Hence, standardized manual chart review is commonly used to validate results [37].

In this study, we aim to use EHRs to evaluate the impact of GLP1 RA, DPP4i, insulin, and sulfonylureas on HF in patients with T2D for whom SGLT2i are unavailable, contraindicated, or unsuccessful in achieving glycemic targets. Considering the stark heterogeneity in patient characteristics associated with HF profile, for example, access to care and insulin resistance [7,38], we focused on the comparison within traditional treatments inducing insulin resistance (insulin vs sulfonylureas) and newer treatments reducing insulin resistance (GLP1 RA vs DPP4i). To characterize potential confounding, we identified EHR features (codes or terms from notes) relevant to T2D through a knowledge network [32] and adjusted for their counts in pretreatment windows by causal estimation [39,40].

## Methods

### Ethical Considerations

The study was approved by the Institutional Review Board at the University of Minnesota with waiver for informed consent (STUDY00018213). Patients who opted out of the use of EHRs for research were excluded from the study, with a waiver for informed consent. The analysis was conducted with limited data hosted in an HIPPA compliant server to minimize the risks to participants’ privacy and confidentiality.

### Study Data

We extracted the data from the EHRs of 201,212 patients who received care at M Health Fairview and had T2D diagnosis codes identified by the *International Classification of Diseases version 9* and *version 10* under the PheWAS catalog PheCode 250.2 [41] (Table S1 in Multimedia Appendix 1). Because some patients with T2D diagnosis codes might not have T2D due to mistakes in documentation or nondiagnosis use of the code for billing, we deployed a multimodal automated phenotyping (MAP) algorithm to further filter for patients likely to have T2D [42]. MAP is a validated, unsupervised algorithm that classifies disease status based on counts of diagnosis codes and mentions in notes for the target disease adjusted by the total number of health care encounters [42,43]. A clinically trained annotator reviewed the disease status of 50 randomly selected patients to validate the accuracy of diagnosis codes and MAP predictions. We chose a 95% specificity cutoff for the MAP T2D filter and defined the phenotyped T2D-positive patients as those with MAP predictions greater than the cutoff. We randomly subsampled 5000 patients from the phenotyped T2D positive patients. We extracted extensive EHR data for this cohort, including all EHR codes (diagnosis, procedure order and result, medication, and laboratory test result) and narrative notes. We deployed a natural language processing (NLP) pipeline [44] to recognize the medical terms in notes compiled in the Unified Medical Language System [45].

### Study Design

We considered 4 groups of medications as interventions, including insulin; sulfonylureas (chlorpropamide, glyburide, glipizide, glimepiride, and tolbutamide); DPP4i (linagliptin, alogliptin, saxagliptin, and sitagliptin); and GLP1 RA (dulaglutide, lixisenatide, albiglutide, exenatide, liraglutide, and semaglutide; Figure S1 in Multimedia Appendix 1). We did not include the following medications approved to treat T2D as interventions of interest in the comparisons: SGLT2i (empagliflozin, canagliflozin, dapagliflozin, ertugliflozin, bexagliflozin, and sotagliflozin) for their established benefits of reducing HF risks [46,47]; and thiazolidinediones for their contraindication in patients with HF [48]. Patients who received SGLT2i prior to treatments of interest were still included in the study, with their past SGLT2i treatment used as a baseline variable. The index date (time 0) of the study was defined as the initiation of the first treatment among the list of interest. Inclusion criteria were patients aged older than 18 years, prior metformin treatment (Figure S2 in Multimedia Appendix 1), established care >365 days prior to index date, and maintained care >30 days after index date (Figure S3 in Multimedia Appendix 1). We identified potential treatment with metformin and subsequent medications of interest by their RxNorm medication codes [49]. The inclusion criterion of previous metformin treatment was based on the long-time role as a typical first-line treatment throughout the observation window in our data [15]. Besides, requiring at least 1 previous treatment may reduce the data leakage issue of baseline T2D characteristics from past treatments at another health institute. To filter out potential spurious codes [50], we required a pair of medication codes for the same ingredient to occur 30 to 180 days apart, reflecting the revolving cycles of established T2D treatments. For treatment identified by this rule, we considered the date of the first medication code in the qualifying pair as the treatment initiation date. Eligible patients were assigned to insulin, sulfonylureas, DPP4i, or GLP1 RA groups based on their treatment after being on metformin. We also extracted the treatment data in the first 5 years of the follow-up to summarize the continuation of metformin or switching to treatments of a different class (Table S2 in Multimedia Appendix 1). We defined established and maintained care as having at least 1 medical encounter occurring more than 365 days prior to the index date and more than 30 days after the index date.

The primary HF outcome in the study was HF-related hospitalization or mortality up to 5 years from the index date. Mortality information was extracted from death records in the EHRs. A clinically trained annotator reviewed the medical charts of eligible patients from the index date to 5 years after to determine the presence of HF-related hospitalization and the corresponding date. We considered the participants censored at the date of their last medical encounter in EHRs.

To measure confounding, we compiled an extensive list of baseline variables. We extracted sex; age at baseline; race; ethnicity; baseline BMI; rural or urban residence; duration of T2D (from the date of first T2D diagnosis code to the index date); duration of metformin treatment (from date of identified metformin initiation to the index date); past HF history determined by the occurrence of HF diagnosis code; past treatment of SGLT2i; and baseline laboratory tests including glycated hemoglobin, low-density lipoprotein, high-density lipoprotein, and total cholesterol. Laboratory tests and BMI values were retrieved within a year before baseline and marked as missing data if no measurement was taken during the year; if more than 1 measurement was available, we selected the one closest to baseline. Missing test values were imputed by the mean of complete cases. In addition to these expert-selected variables, we adjusted for a broad list of EHR features related to T2D or CVD, according to the knowledge network Online Narrative and Codified Feature Search Engine [32]. The full list consisted of 251 diagnosis PheCodes [41], 55 laboratory test Logical Observation Identifier Names and Codes [51], 43 medication RXNORM codes [49], 13 procedure Clinical Classification Software codes [52], and 386 NLP concept unique identifiers [45]. These are all common ontologies rolled up from raw EHR codes [31]. To better characterize the baseline CVD risks, we additionally summarized baseline CVD medications according to their mechanisms (Figure S4 in Multimedia Appendix 1). We summarized these EHR features by counting in 2 temporal windows: more than 1 year prior to the index date for medical history and within 1 year prior to the index date for recent medical conditions (Figure S3 in Multimedia Appendix 1). We filtered out the variables that occurred in fewer than 5% (47/939) of the patients.

### Statistical Analysis

For each comparison, we estimated 2 treatment effect parameters: risk differences in 5-year HF-related hospitalization or death and the ratio in 5-year non-HF survival rate. We applied doubly robust estimation methods for both treatment effect parameters that optimally integrate outcome regression and inverse propensity score weighting to achieve robustness to model specifications [40,53]. To select confounders from the large number of candidates, we used the adaptive least absolute shrinkage and selection operator to estimate the propensity score model, logistic outcome regression model for risk difference, and additive hazards outcome regression model for no-HF survival ratio [54]. We accounted for censoring in risk difference analysis by inverse probability of censoring weighting, assuming independent censoring. We used bootstrap to calculate the SD of the estimates and normal approximation to calculate the 95% CIs. Our primary analysis is an intent-to-treat style that ignores the postindex treatment switching. As a sensitivity analysis, we performed a per-protocol style analysis that excluded all patients who switched to another treatment class.

All analyses were conducted in R (version 4.3.1; R Foundation for Statistical Computing). *P* values less than .05 were considered significant.

## Results

### Study Cohort

**Figure 1.**
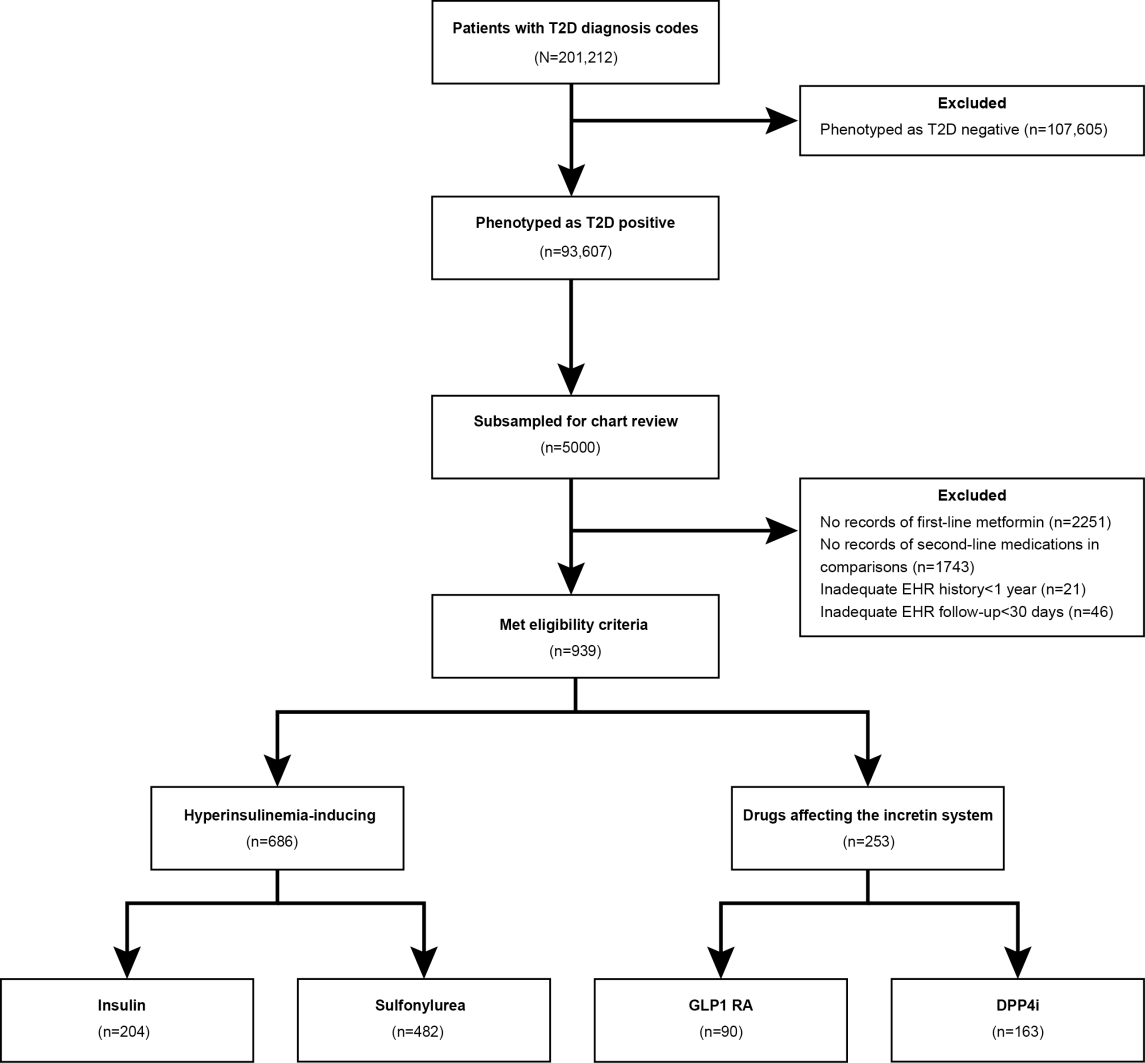
Construction of study cohort. DPP4i: dipeptidyl peptidase-4 inhibitors; EHR: electronic health record; GLP1 RA: glucagon-like peptide-1 receptor agonists; T2D: type 2 diabetes.

A total of 93,607 patients were phenotyped as T2D positive using the MAP algorithm, from which we subsampled 5000 patients for validation of their data through chart review. A subset of 939 patients satisfied all eligibility criteria for comparisons in HF outcomes, including 204 who received insulin, 482 who received sulfonylurea, 90 who received GLP1 RA, and 163 who received DPP4i (Figure 1). Among the 939 patients included in the comparisons, 411 (43.8%) were female and 528 (56.2%) were male; median baseline age was 59.4 (IQR 52.1‐67.9) years; 922 (98.2%) were urban residents and 17 (1.8%) were rural residents; 9 (1%) were American Indian or Alaska Native, 65 (6.9%) were Asian, 81 (8.6%) were Black, 1 (0.1%) was Pacific Islander, 780 (83.1%) were White, and 3 (0.3%) were of more than one race; 19 (2%) were Hispanic; median duration of diabetes was 3.9 (IQR 1.4‐7.0) years; median duration of metformin use was 0.7 (IQR 0.0‐2.6) years; baseline hemoglobin A_1c_ was 8.2% (IQR 7.1%‐9.2%); 49 (5.2%) had preexisting HF (Table 1); and 6 (0.6%) had used SGLT2i previously (Table S2 in Multimedia Appendix 1). From the manual chart review, we identified HF-related hospitalization for 132 (14.1%) patients during the follow-up.

**Table 1.** Characteristics of eligible patients included in the study (N=939).

Variable or level	Summary
Age (y), median (IQR)	59.4 (52.1‐67.9)
Sex, n (%)	
Female	411 (43.8)
Male	528 (56.2)
Residence, n (%)	
Urban	922 (98.2)
Rural	17 (1.8)
Race, n (%)	
American Indian or Alaska Native	9 (1)
Asian	65 (6.9)
Black	81 (8.6)
Native Hawaiian or other Pacific Islander	1 (0.1)
White	780 (83.1)
More than 1 race	3 (0.3)
Ethnicity, n (%)	
Hispanic	19 (2)
Not Hispanic	920 (98)
Diabetes duration (y), median (IQR)	3.9 (1.4‐7.0)
Duration of metformin treatment (y), median (IQR)	0.7 (0.0‐2.6)
Hemoglobin A_1c_ level (%), median (IQR)	8.2 (7.1‐9.2)
Preexisting heart failure, n (%)	49 (5.2)

### Confounding Adjustments

**Figure 2.**
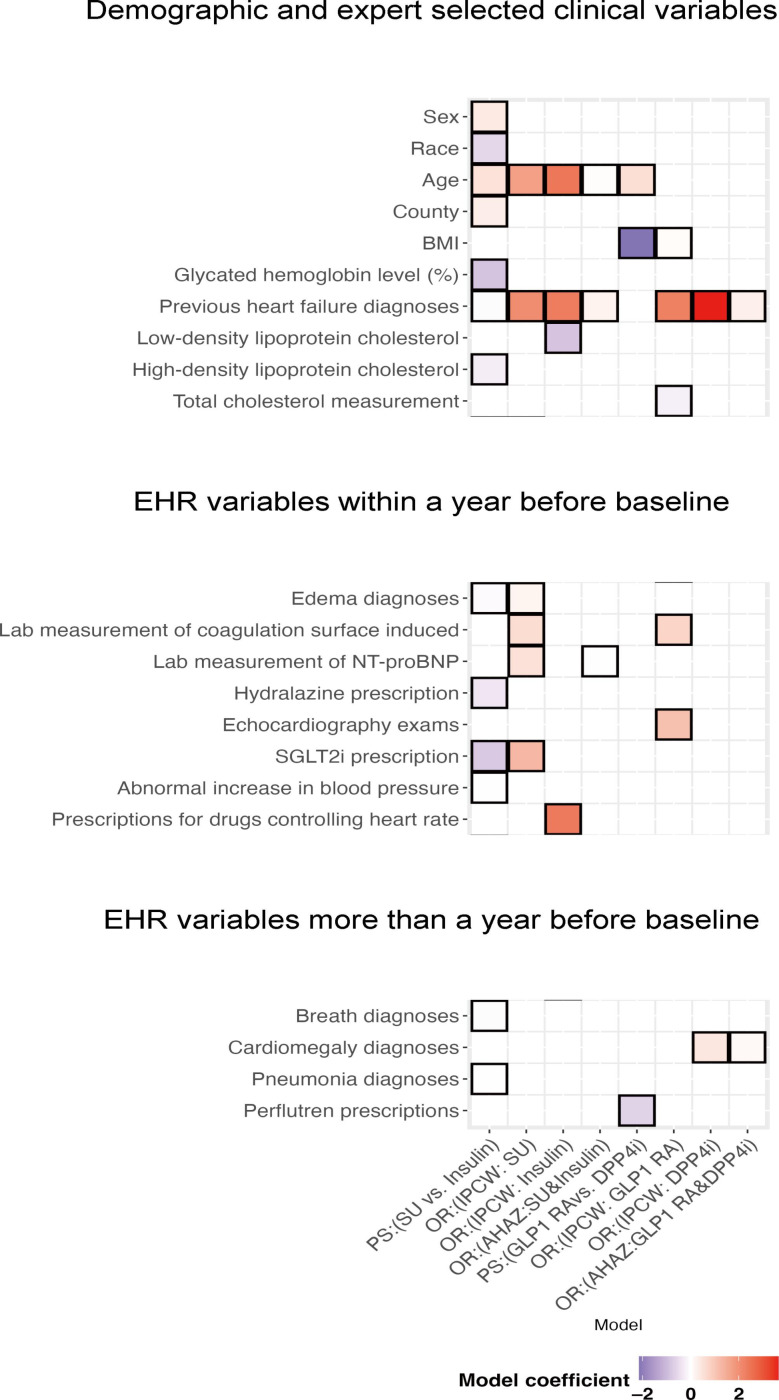
Adjusted confounding in analysis. EHR: electronic health record; SGLT2i: sodium-glucose cotransporter 2 inhibitors.

Besides standard confounding factors, such as age, BMI, and preexisting HF, our analysis identified and adjusted for additional confounding factors among the variables compiled form knowledge networks, including a recent diagnosis of edema, recent prescriptions of SGLT2i, past prescriptions of perflutren, and a contrast agent used for heart ultrasound imaging indicative for baseline cardiovascular conditions (Figure 2). After adjusting for the propensity score, all demographic and clinical variables were balanced between the treatment groups in comparison (Tables S3 and S4 in Multimedia Appendix 1).

### Insulin Versus Sulfonylureas

**Figure 3.**
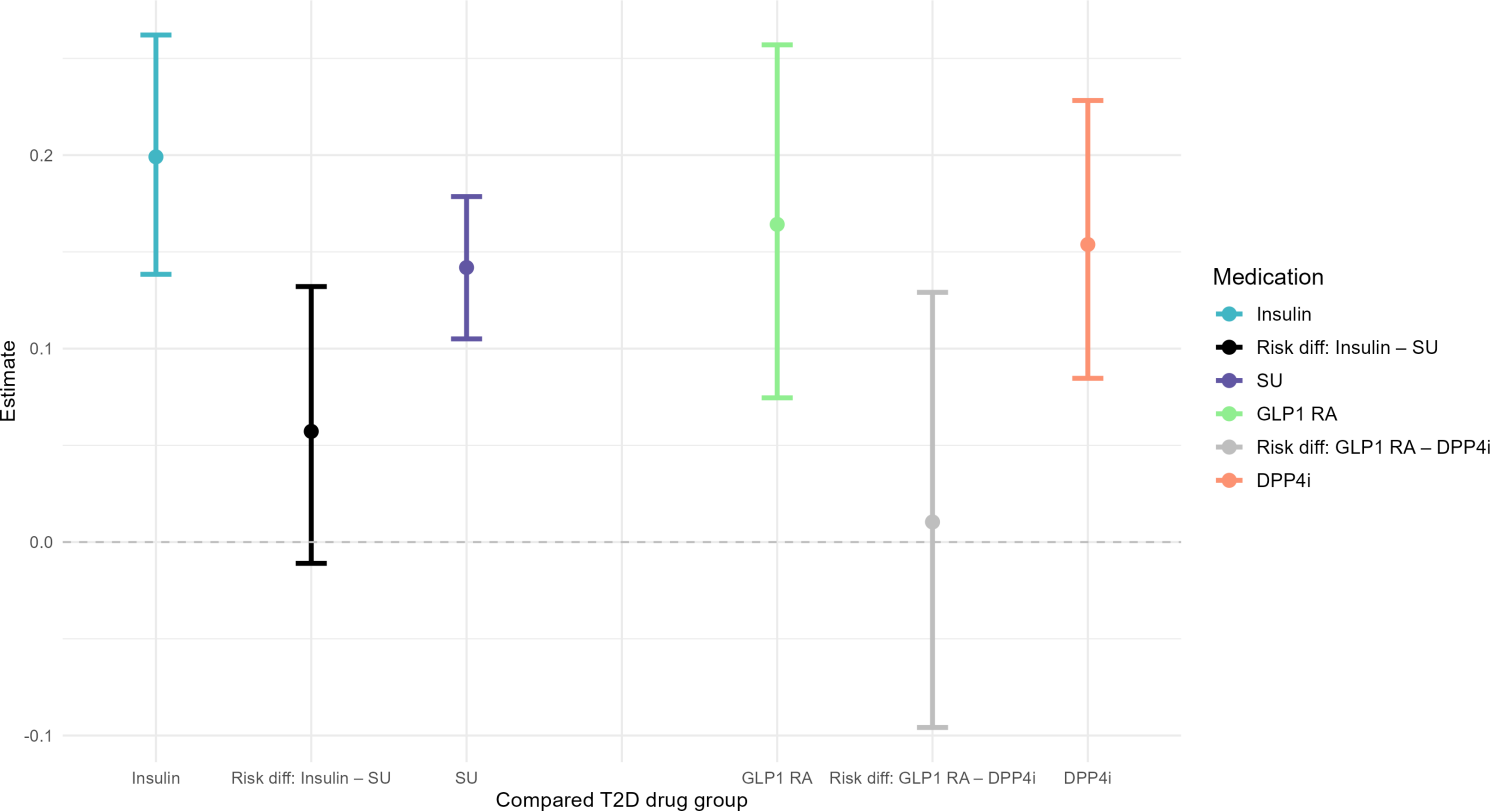
Analysis results on risk differences. DPP4i: dipeptidyl peptidase-4 inhibitors; GLP1 RA: glucagon-like peptide-1 receptor agonists; SU: sulfonylureas; T2D: type 2 diabetes.

**Figure 4.**
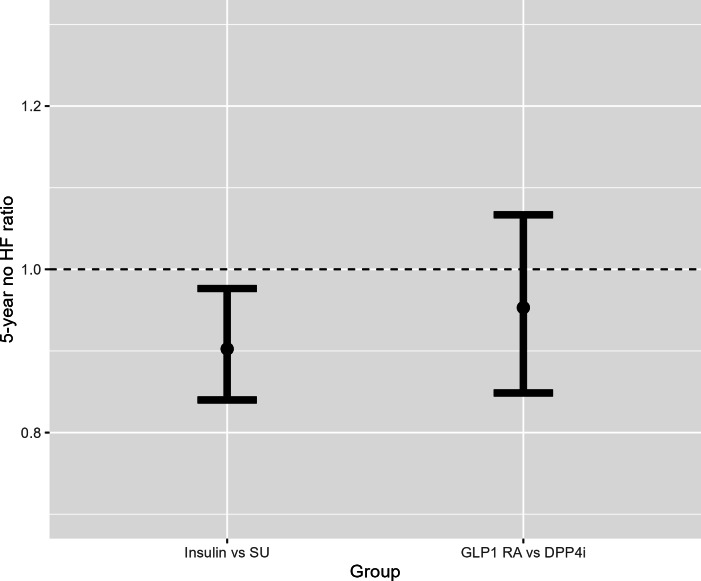
Analysis results on HF-free survival ratios. DPP4i: dipeptidyl peptidase-4 inhibitors; GLP1 RA: glucagon-like peptide-1 receptor agonists; HF: heart failure; SU: sulfonylureas.

After adjusting for confounding and censoring, we estimated that 0.199 (95% CI 0.138‐0.262) of patients who received insulin had a HF-related medical encounter within 5 years, compared to 0.142 (95% CI 0.105‐0.179) of patients who received sulfonylureas, with a risk difference 0.057 (95% CI –0.010 to 0.132; *P=*.11; Figure 3). We estimated that the 5-year HF-free survival ratio for insulin versus sulfonylureas was 0.902 (95% CI 0.840‐0.976; *P=*.01; Figure 4).

### GLP1 RA Versus DPP4i

After adjusting for confounding and censoring, we estimated that 0.164 (95% CI 0.075‐0.257) of patients who received GLP1 RA agonists had a HF-related medical encounter within 5 years, compared to 0.154 (95% CI 0.085‐0.228) of patients who received DPP4i, with a risk difference 0.010 (95% CI –0.096 to 0.129; *P=*.85; Figure 3). We estimated that the 5-year HF-free survival ratio for GLP1 RA versus DPP4i was 0.953 (95% CI 0.849‐1.067; *P=*.40; Figure 4).

### Secondary and Sensitivity Analyses

We present the results of the per-protocol analysis and a comparison of treatments designed to increase versus reduce insulin resistance in Multimedia Appendix 1. The exclusion of patients who switched treatment resulted in a decrease in the estimated HF-related hospitalization for GLP1 RA, DPP4i, and sulfonylurea, resulting in a significant difference for sulfonylurea versus insulin (risk difference 0.103, 95% CI 0.004‐0.197; *P=*.04). We did not observe significant differences for all other analyses.

## Discussion

### Principal Findings

In this observational study, we found that while patients receiving sulfonylureas had better HF-free survival when compared to those receiving insulin by risk ratio, the risk difference was not significant. Compared to the risk difference analysis, the HF-free survival ratio analysis additionally used the timing of HF-related health care encounters within 5 years, which potentially increased its testing power. Our comparisons of GLP1 RA versus DPP4i failed to detect significant differences in 5-year HF outcomes with the current sample size.

Our findings concerning insulin and sulfonylureas are consistent with previous studies, which examined all CVDs, and not HF specifically. Insulin was reported to be associated with a higher rate of CVD events [55] compared to sulfonylureas as a second-line treatment. A previous study [56] showed that patients on sulfonylureas and insulin had a higher CVD risk than those on GLP1 RA, DPP4i, and SGLT2i, but it found no significant difference for patients on sulfonylureas and insulin. Both studies used composite CVD outcomes as opposed to HF exacerbations specifically. They are particularly important because frequent exacerbations are the most costly and burdensome aspect of the disease [7,8]; therefore, finding factors that make them less frequent is vital for the health care system.

There were no significant differences in HF outcomes for GLP1 RA and DPP4i, while a trial emulation study reported that patients on GLP1 RA had a lower CVD risk than those on DPP4i [27]. However, their outcome of interest was major adverse cardiovascular events, and not specifically time-to-HF exacerbation. A 2021 systematic review aiming to summarize the effect of GLP-1 RAs compared to DPP-4i showed conflicting results, with one study suggesting the benefit of GLP-1 RAs and another favoring DPP-4i [57], while a 2022 meta-analysis showed a similar risk of HF-related hospitalizations [58].

We included a broader real-world patient population consisting of both patients with and without preexisting HF, while most existing studies targeted a high-risk subpopulation with prior CVDs. Nevertheless, the rates of HF-related hospitalizations in our study were consistent with those found in the literature. HF is associated with an over 50% five-year mortality rate [59], and a 2023 meta-analysis showed a 35.7% (95% CI 27.1%-44.9%) 1-year readmission rate in patients previously admitted for HF [60]. Moreover, a local study conducted in Minnesota showed that among 1077 patients with HF followed over a mean period of 4.7 years, there were 713 HF-related hospitalizations, and 32.3% of the cohort were hospitalized due to HF at least once [61]. Among patients with T2D in the Empagliflozin Cardiovascular Outcome Event (EMPA-REG OUTCOME) randomized clinical trial of empagliflozin, the HF hospitalization rates were lower and equaled 2.7% (empagliflozin) or 4.1% (placebo) over a median follow-up time of 3.1 years [19]. However, these were patients who fulfilled the inclusion criteria and not the real-world population. Studies included in the meta-analysis conducted by Xu et al [58] reported that HF-related hospitalization rates in patients with T2D were between 0.6% and 8.7% per year [11-13] but increased to almost 30% per year if only patients with a previous HF diagnosis were included [11].

The most important predictors in HF outcome models were preexisting HF and the N-terminal pro-B-type natriuretic peptide laboratory tests, a biomarker for HF [62], which are clearly strong predictors of a future HF exacerbation. Propensity score models contained numerous other variables, including many EHR-derived, NLP-derived, and hierarchical medication structure variables. This shows that treatment assignment is a complicated process, which depends on many factors, and obtaining credible results of the final analysis requires state-of-the-art approaches to EHR data. There is no single straightforward T2D treatment regime sequence [18], and in some patients, clinical practitioners choose between insulin and sulfonylureas. This is why a comprehensive comparison between T2D drugs is crucial for clinicians to guide their decisions. In light of our findings, sulfonylureas might be associated with lower risks of HF-related hospitalization compared to insulin, but this needs to be validated by further studies with larger sample sizes and less confounding designs.

### Limitations

First, as in most observational studies, unadjusted confounding may exist and compromise the conclusions. Second, the sample sizes were limited by our capacity to manually annotate clinical data and may lead to underpowered analyses. After applying eligibility, the resulting sample sizes could only detect >6.7% risk difference for insulin versus sulfonylureas, and >10.6% risk difference or >12% relative risks for GLP1 RA versus DPP4i. While our subsampling for annotation was completely random and supposedly representative, the eligibility criteria may have introduced selection bias due to imprecise data. We also could not identify patients assigned to combination therapies (eg, metformin and GLP1 RA) based on prescription codes alone. As artificial intelligence language models advance, we envision that artificial intelligence–assisted data abstraction will significantly increase the availability of high-quality data. Furthermore, the data annotated from this study may facilitate the training and validation of these future tools. Third, our intent-to-treat style primary analysis only considered the point decision of treatment with medication initiated after metformin, which did not account for subsequent treatment switching nor investigate sequential treatment strategies. Interpretation of intent-to-treat style analysis is dependent on the subsequent treatment patterns and may not generalize to other institutions or future times [63]. While our sensitivity analysis produced generally lower HF-related hospitalization rates, it may be subject to selection bias if treatment switching was informative for poor HF outcomes. Previous studies [64,65] have proposed the use of composite outcomes involving treatment switching or discontinuation, but the relatively high rate of treatment switching in this study would dominate the composite outcome and deviate substantially from our focus on severe HF outcomes. Similarly, postindex SGLT2i or metformin use may impact the HF outcomes and is difficult to adjust for due to its likely informativeness with HF outcomes. While we observed heterogeneity among treatment groups, the higher postindex SGLT2i use (GLP1 RA and insulin) is associated with a higher estimated HF-related hospitalization (Table S2 in Multimedia Appendix 1; Figure 3). These questions can be revisited once corresponding statistical methods are developed. Finally, the study only included patients from a single institute and lacked the validation of generalizability. Multi-institutional collaboration through research networks may be considered in the future [65].

### Conclusions

In conclusion, we created a T2D study cohort from EHRs with annotated clinical data and compared the 5-year HF outcomes of insulin versus sulfonylureas and GLP1 RA versus DPP4i. After adjustment for confounding, we found that sulfonylureas were associated with a reduced risk for HF-related hospitalization compared to insulin, while we could not detect a significant difference for HF outcomes in patients treated with GLP1 RA and DPP4i with the current sample size. Our findings provided additional evidence to guide clinical decisions for managing T2D.

## Supplementary material

10.2196/85083 Multimedia Appendix 1 Secondary and sensitivity analyses results and supplementary figures and tables.

## Abbreviations

CVD: cardiovascular disease
DPP4i: dipeptidyl peptidase-4 inhibitors
EHR: electronic health record
GLP1 RA: glucagon-like peptide-1 receptor agonists
HF: heart failure
MAP: multimodal automated phenotyping
NLP: natural language processing
SGLT2i: sodium-glucose cotransporter 2 inhibitors
T2D: type 2 diabetes mellitus
