# Supplementary material for: Antidiabetic Drug Associations With Heart Failure Outcomes: Real-World Evidence Study Using Electronic Health Records
Source: JMIR Diabetes. 2026 Apr 15;11:e85083. doi: 10.2196/85083 (PMC13082573; doi:10.2196/85083)
Supplement: Multimedia Appendix 1 [file diabetes-v11-e85083-s001.docx]

**SUPPLEMENTARY MATERIAL**

**Results S1. Secondary and Sensitivity Analysis.**

Per-protocol style analysis

After excluding patients who switched to treatment of a different class from their initial treatment at index, we obtained a subset of 574 patients satisfying all eligibility criteria for comparisons in HF outcomes, including 127 received insulin, 289 received sulfonylurea, 44 received GLP1 RA, and 114 received DPP4i. Among the patient included in the comparisons, 240 (41.8%) were female, 334 (58.2%) were male; median baseline age 60.8years (inter-quartile range [IQR] 53.1 – 69.91; 564 (98.3%) were urban residents, 10 [1.7%] were rural residents; 5 (0.9%) were American Indian or Alaska Native, 38 (6.6%) were Asian, 46 (8.0%) were black, 0 (0.0%) was Pacific Islander, 484 (84.3%) were white, 1 (0.2%) were of more than one race; 13 (2.3%) were Hispanic; median diabetes duration 4.2 years (IQR 1.6 – 7.3); median duration of metformin 1 years (IQR 0.1 – 3.1); baseline glycated hemoglobin 8.1% (IQR 7.0% – 9.0%); 35 (6.1%) had pre-existing HF.

After adjusting for confounding and censoring, we estimated that 0.219 (95% CI 0.135 – 0.310) of patients received insulin had HF related medical encounter within 5 years, compared to 0.117 (95% CI 0.078 – 0.166) of patients received sulfonylureas, with a risk difference 0.103 (95% CI 0.004– 0.197, *P=*.04) (**Figure S5**). We estimated the 5-year HF-free survival ratio for insulin vs sulfonylureas was 0.916 (95% CI 0.827 – 1.004, *P=*.06) (**Figure S6**).

After adjusting for confounding and censoring, we estimated that 0.077 (95% CI 0.006 – 0.149) of patients received GLP1 RA had HF related medical encounter within 5 years, compared to 0.136 (95% CI 0.057 – 0.232) of patients received DPP4i, with a risk difference -0.059 (95% CI -0.157 – 0.026, *P=*.21) (**Figure S5**). We estimated the 5-year HF-free survival ratio for GLP1 RA vs DPP4i was 1.081 (95% CI 0.903 – 1.269, *P=*.40) (**Figure S6**).

Increase vs reduce insulin resistance

After adjusting for confounding and censoring, we estimated that 0.159 (95% CI 0.123 – 0.208) of patients received DPP4i or GLP1 RA (reduce insulin resistance) had HF related medical encounter within 5 years, compared to 0.149 (95% CI 0.091 – 0.221) of patients received insulin or sulfonylureas (increase insulin resistance), with a risk difference 0.011 (95% CI -0.069 – 0.081, *P=*.80). We estimated the 5-year HF-free survival ratio for increase vs reduce insulin resistance was 1.015 (95% CI 0.945 – 1.093, *P=*.69).

**Figure S1. T2D medications and their groups.**


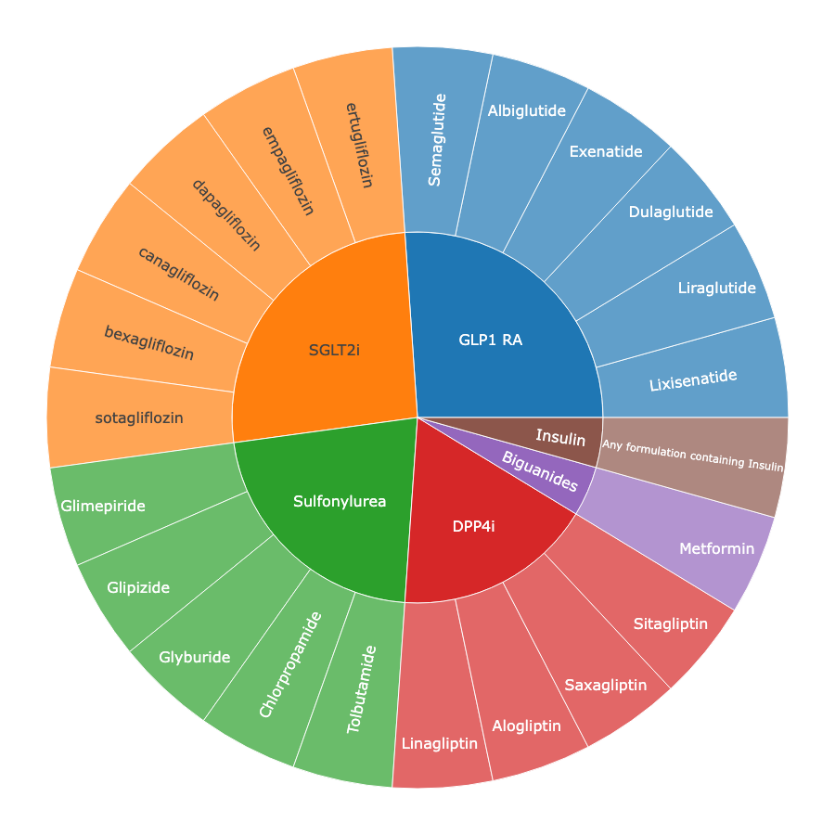


**Figure S2. Examples for eligibility criteria on the orders of treatments.**


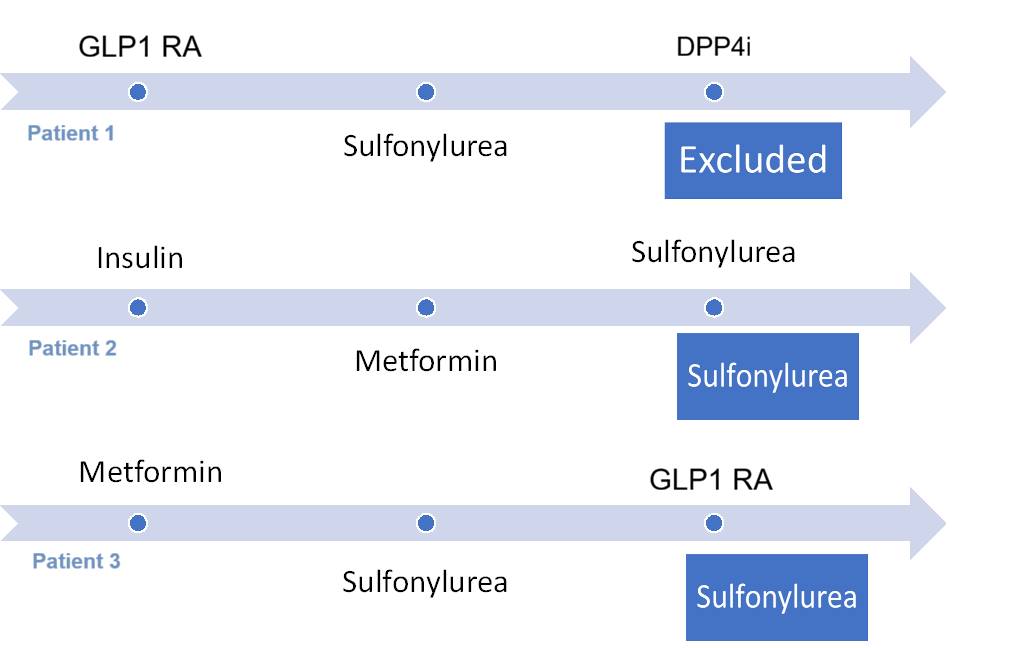


**Figure S3. Eligibility criteria on relative timeline and study periods for covariates and outcomes.
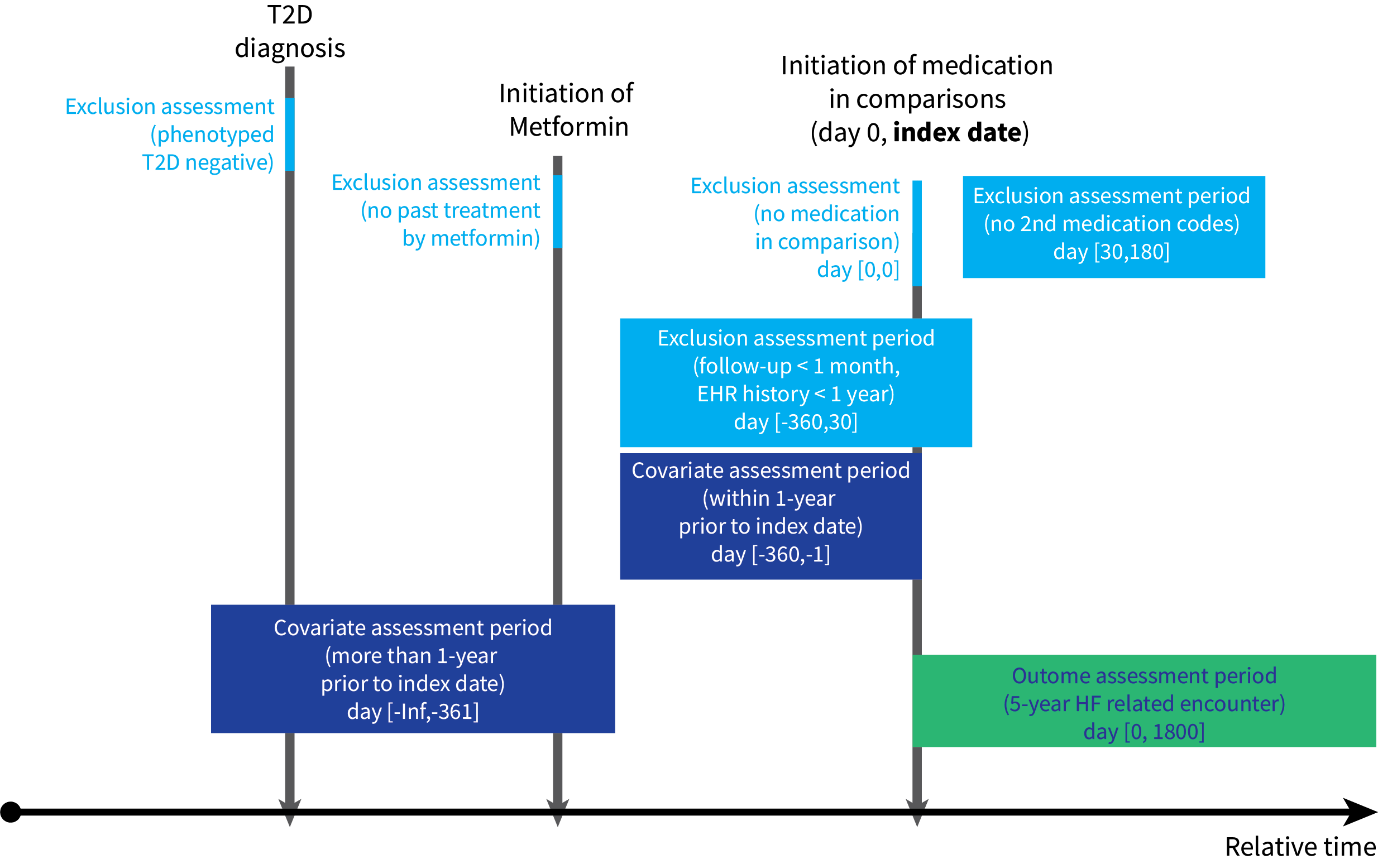
**

**Figure S4. CVD medications and their groups.**

**
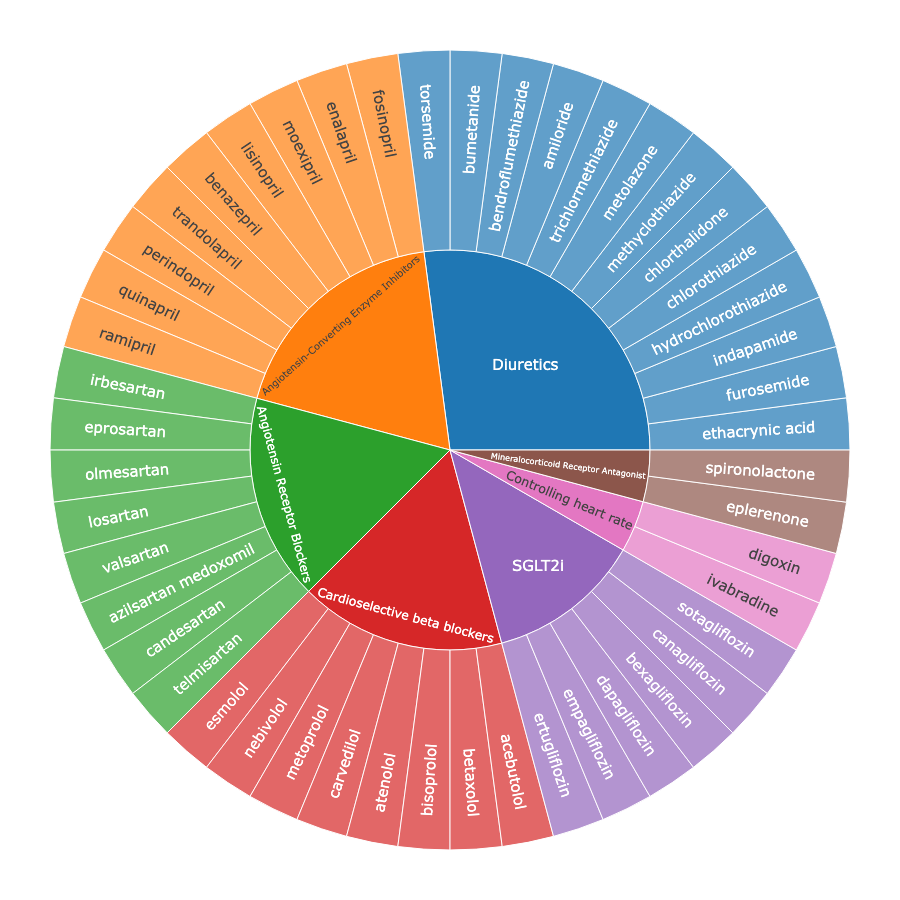
**

**Figure S5. Per-protocol style analysis results on risk differences.** Adjusted average 5-year HR rates for insulin, sulfonylureas, GLP1 RA, DPP4i groups and risk differences insulin vs sulfonylureas, GLP1 RA vs DPP4i with 95% confidence intervals.

**
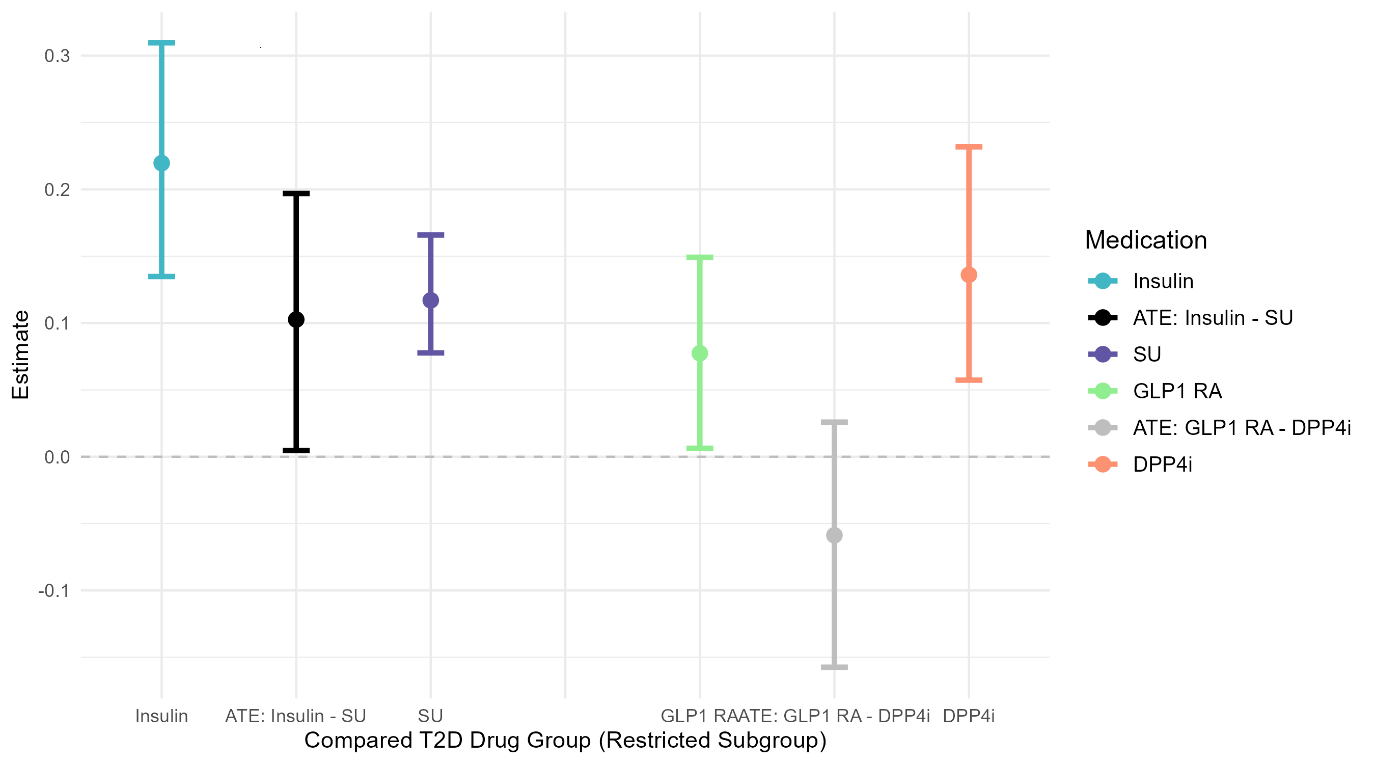
**

**Figure S6. Per-protocol style analysis results on HF-free survival ratios.** Adjusted 5-year HF-free survival ratio for sulfonylureas vs. Insulin and GLP1 RA VS. DPP4i.

**
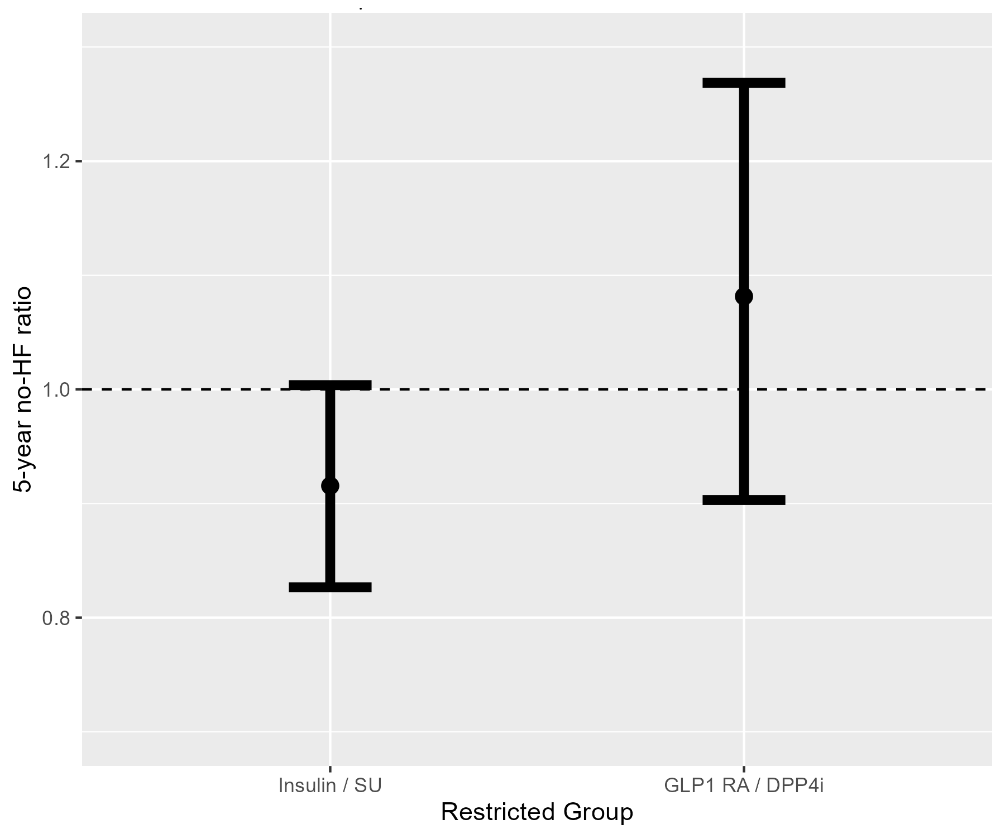
**

**Table S1. List of ICD Codes for PheCode 250.2 for T2D.**

| **Codes** | **Descriptions** |
| --- | --- |
| ICD-9:250 | type II diabetes mellitus [non-insulin dependent type] [NIDDM type] [adult-onset type] or unspecified type, not stated as uncontrolled, without mention of complication |
| ICD-9:250.02 | type II diabetes mellitus [non-insulin dependent type] [NIDDM type] [adult-onset type] or unspecified type, uncontrolled, without mention of complication |
| ICD-9:250.2 | Diabetes mellitus, type II [non-insulin dependent type] [NIDDM type] [adult-onset type] or unspecified type with hyperosmolarity, not stated as uncontrolled |
| ICD-9:250.22 | Diabetes mellitustype II [non-insulin dependent type] [NIDDM type] [adult-onset type] or unspecified type with hyperosmolarity, uncontrolled |
| ICD-9:250.3 | Diabetes mellitus, type II [non-insulin dependent type] [NIDDM type] [adult-onset type] or unspecified type, not stated as uncontrolled |
| ICD-9:250.32 | Diabetes mellitus with other coma, type II [non-insulin dependent type] [NIDDM type] [adult-onset type] or unspecified type, uncontrolled |
| ICD-9:250.8 | Diabetes mellitus type II [non-insulin dependent type] [NIDDM type] [adult-onset type] or unspecified type, not stated as uncontrolled, with other specified manifestations |
| ICD-9:250.82 | Diabetes mellitus type II [non-insulin dependent type] [NIDDM type] [adult-onset type] or unspecified type, uncontrolled, with other specified manifestations |
| ICD-9:250.9 | Diabetes mellitus type II [non-insulin dependent type] [NIDDM type] [adult-onset type] or unspecified type, not stated as uncontrolled, with unspecified complication |
| ICD-9:250.92 | Diabetes mellitus type II [non-insulin dependent type] [NIDDM type] [adult-onset type] or unspecified type, uncontrolled, with unspecified complication |
| ICD-10:E11.6 | Type 2 diabetes mellitus with other specified complications |
| ICD-10:E11.64 | Type 2 diabetes mellitus with hypoglycemia |
| ICD-10:E11.649 | Type 2 diabetes mellitus with hypoglycemia without coma |
| ICD-10:E11.641 | Type 2 diabetes mellitus with hypoglycemia with coma |
| ICD-10:E11.630 | Type 2 diabetes mellitus with periodontal disease |
| ICD-10:E11.69 | Type 2 diabetes mellitus with other specified complication |
| ICD-10:E11.620 | Type 2 diabetes mellitus with diabetic dermatitis |
| ICD-10:E11.610 | Type 2 diabetes mellitus with diabetic neuropathic arthropathy |
| ICD-10:E11.65 | Type 2 diabetes mellitus with hyperglycemia |
| ICD-10:O24.11 | Pre-existing type 2 diabetes mellitus, in pregnancy |
| ICD-10:E11.61 | Type 2 diabetes mellitus with diabetic arthropathy |
| ICD-10:E11.628 | Type 2 diabetes mellitus with other skin complications |
| ICD-10:E11.0 | Type 2 diabetes mellitus with hyperosmolarity |
| ICD-10:E11 | Type 2 diabetes mellitus |
| ICD-10:E11.621 | Type 2 diabetes mellitus with foot ulcer |
| ICD-10:E11.8 | Type 2 diabetes mellitus with unspecified complications |
| ICD-10:E11.63 | Type 2 diabetes mellitus with oral complications |
| ICD-10:E11.9 | Type 2 diabetes mellitus without complications |
| ICD-10:E11.01 | Type 2 diabetes mellitus with hyperosmolarity with coma |
| ICD-10:E11.00 | Type 2 diabetes mellitus with hyperosmolarity without nonketotic hyperglycemic-hyperosmolar coma (NKHHC) |
| ICD-10:E11.62 | Type 2 diabetes mellitus with skin complications |
| ICD-10:E11.622 | Type 2 diabetes mellitus with other skin ulcer |
| ICD-10:E11.638 | Type 2 diabetes mellitus with other oral complications |
| ICD-10:E11.618 | Type 2 diabetes mellitus with other diabetic arthropathy |

**Table S2. Summary of baseline SGLT2i treatment and switching to new class of treatment during the first 5 years of follow-up.**

| **Variable** | **DPP4i (n=166)** | **GLP1 RA (n=92)** | **Insulin (n=231)** | **SU (n=488)** |
| --- | --- | --- | --- | --- |
| **Treatment prior to index date** |  |  |  |  |
| SGLT2i use (count [%]) | 3 [1.8%] | 2 [2.2%] | 0 [0.0%] | 1 [0.2%] |
| **Treatment in 5-year follow-up** |  |  |  |  |
| SGLT2i use (count [%]) | 17 [10.2%] | 14 [15.2%] | 24 [10.4%] | 42 [8.6%] |
| Metformin use (count [%]) | 10 [6.0%] | 21 [22.8%] | 42 [18.2%] | 130 [26.6%] |
| **Switching in 5-year follow-up** |  |  |  |  |
| DPP4i use (count [%]) | – | 23 [25.0%] | 50 [21.6%] | 83 [17.0%] |
| GLP1 RA use (count [%]) | 2 [1.2%] | – | 16 [6.9%] | 51 [10.5%] |
| Insulin use (count [%]) | 37 [22.3%] | 23 [25.0%] | – | 122 [25.0%] |
| SU use (count [%]) | 22 [13.3%] | 21 [22.8%] | 30 [13.0%] | – |
| No switching (count [%]) | 116 [69.9%] | 43 [46.7%] | 147 [63.6%] | 294 [60.2%] |

**Table S3. Balancing assessment of demographic and baseline clinical characteristics for insulin vs SU.**

|  | Observed | | | Inverse PS Weighted | | |
| --- | --- | --- | --- | --- | --- | --- |
|  | **Insulin** | **SU** | **p-value** | **Insulin** | **SU** | **p-value** |
| Age (median [IQR]) | 59.3 [51.2 – 68.8] | 60.6 [53.0 – 69.3] | .317 | 60.0 [52.1 – 68.6] | 60.2 [53 – 69.7] | 1.00 |
| Female (count [%]) | 105 [51.5%] | 190 [39.4%] | .005 | 315 [45.9%] | 292 [42.5%] | .38 |
| Urban (count [%]) | 202 [99%] | 472 [97.9%] | .50 | 674 [98.2%] | 673 [98.1%] | .89 |
| Hispanic (count [%]) | 7 [3.4%] | 4 [0.8%] | .03 | 15 [2.2%] | 7 [0.9%] | .18 |
| White (count [%]) | 149 [73%] | 410 [85.1%] | <.001 | 553 [80.6%] | 562 [82%] | .64 |
| Diabetes duration [years] (median [IQR]) | 3.5 [0.9 – 6.5] | 3.9 [1.5 – 6.8] | .05 | 3.6 [0.9 – 6.7] | 4.1 [1.6 – 7.2] | .89 |
| Duration of metformin [years] (median [IQR]) | 0.5 [0 – 1.9] | 0.7 [0 – 2.5] | .06 | 0.5 [0 – 1.8] | 0.6 [0 – 2.5] | .85 |
| Glycated hemoglobin level [%] (median [IQR]) | 8.4 [7.1 – 10.2] | 8.1 [7.1 – 8.9] | .002 | 8.4 [7.1 – 10.3] | 8.1 [7.2 – 8.8] | 1.00 |
| BMI (median [IQR]) | 32.8 [29 – 38.9] | 33.4 [29.2 – 38.4] | .66 | 33.1 [29.7 – 38.8] | 33.5 [29 – 38.7] | 1.00 |
| Pre-existing HF (count [%]) | 10 [4.9%] | 16 [3.3%] | .44 | 31 [4.5%] | 24 [3.6%] | .53 |

* Tests for the univariate association between covariates and treatment groups by two-sample Kolmogorov-Smirnov test for observed data.

† Tests for the univariate association between covariates and treatment groups by weighted two-sample Kolmogorov-Smirnov test with inverse propensity scores.

**Table S4. Balancing assessment of demographic and baseline clinical characteristics for GLP1 RA vs DPP4i.**

|  | Observed | | | Inverse PS Weighted | | |
| --- | --- | --- | --- | --- | --- | --- |
|  | **GLP1 RA** | **DPP4i** | **p-value** | **GLP1 RA** | **DPP4i** | **p-value** |
| Age (median [IQR]) | 56.3 [49.7 – 63.6] | 60.1 [53.8 – 67.1] | .02 | 56.1 [49.6 – 64.8] | 60.1 [53.7 – 67.2] | .93 |
| Female (count [%]) | 76 [46.6%] | 40 [44.4%] | .84 | 116 [45.9%] | 120 [47.5%] | .79 |
| Urban (count [%]) | 159 [97.6%] | 89 [98.9%] | .79 | 246 [97.2%] | 247 [97.7%] | .80 |
| Hispanic (count [%]) | 7 [4.3%] | 1 [1.1%] | .31 | 11 [4.3%] | 1 [0.6%] | .06 |
| White (count [%]) | 138 [84.7%] | 69 [76.7%] | .16 | 215 [85.1%] | 207 [81.8%] | .48 |
| Diabetes duration [years] (median [IQR]) | 5 [1.8 – 8.1] | 4 [1.8 – 6.9] | .45 | 5.1 [2 – 8.1] | 3.8 [1.1 – 6.2] | .47 |
| Duration of metformin [years] (median [IQR]) | 1.4 [0.3 – 3.4] | 0.8 [0 – 3.4] | .045 | 1.2 [0.2 – 3.4] | 0.7 [0 – 2.8] | .45 |
| Glycated hemoglobin level [%] (median [IQR]) | 8.1 [7.1 – 9] | 8.1 [7.1 – 9] | .92 | 8 [7 – 9.2] | 8.2 [7.1 – 9] | 1.00 |
| BMI (median [IQR]) | 36.9 [31.2 – 42.2] | 32.2 [27.4 – 37.8] | .001 | 36.9 [31.3 – 41.9] | 32.1 [27.3 – 37.8] | 1.00 |
| Pre-existing HF (count [%]) | 14 [8.6%] | 9 [10%] | .88 | 21 [8.3%] | 24 [9.6%] | .71 |

* Tests for the univariate association between covariates and treatment groups by two-sample Kolmogorov-Smirnov test for observed data.

† Tests for the univariate association between covariates and treatment groups by weighted two-sample Kolmogorov-Smirnov test with inverse propensity scores.
